# The right eye for fixations: Eye asymmetries modulate gaze patterns towards speakers

**DOI:** 10.3758/s13423-026-02892-w

**Published:** 2026-03-11

**Authors:** Desiderio Cano Porras, Max M. Louwerse

**Affiliations:** Department of Computational Cognitive Science, Tilburg University, Tilburg, The Netherlands 5037AB

**Keywords:** Eye gaze, Eye contact, Eye asymmetry, Communication, Social function

## Abstract

Eye contact is critical in face-to-face social interactions. Prior research has shown that dialog partners primarily focus on the eyes of the speaker both when the speaker is speaking and, particularly, when the speaker is not. These findings are compatible with the communicative but specifically the social function of eye contact. This is in line with animal cognition literature showing that animals with more eye sclera tend to be more social. Several human studies have reported a left perceptual bias in eye contact, with the listener focusing on the right side of the speaker’s face. Some studies attributed this bias to hemispheric specialization. Here two eye-tracking experiments using human and virtual human speakers confirmed a systematic bias towards the right eye of the speaker. Findings, however, are modulated by the amount of sclera and communicative events. Larger eyes (more sclera visibility) attracted more fixations, so that faces with larger *left* eyes do not systematically induce the left perceptual bias to the right side of the face. Moreover, a difference in fixations on the left or right eye of the speaker was found depending on whether somebody was speaking or not. Our results are consistent with the left perceptual bias, but suggest the bias is not solely perceptual. Instead, our findings suggest the social function in eye contact modulates the bias towards the right eye. Face-scanning behavior emerges as an unfolding dynamic shaped by a flow of social and communicative action ladders. These findings shed light on the most fundamental aspects of human communicative behavior.

## Introduction

Not looking at the eyes of our conversational partner in face-to-face conversations is awkward, yet the same is true for staring at the other person’s eyes incessantly. Preferred duration of eye contact has been reported to be around 3 s (Binetti et al., 2016; Mayrand et al., 2023). Eye contact has shown to be critical for human interactions, so much so that our gaze usually anchors towards the eyes in face-to-face conversations (Cano Porras & Louwerse, 2025). Gaze fixations towards the mouth increase particularly during speech-perception tasks and when looking at someone talking (Buchan et al., 2007; Frank et al., 2012; Viktorsson et al., 2023), but the eyes keep attracting attention. Making less eye contact when the speaker is speaking, and more eye contact when the speaker is not, supports the social function for eye gaze in addition to its communicative function (Hoffman & Haxby, 2000; Senju & Johnson, 2009; Wicker et al., 2003).

According to the action ladders of communication proposed by Clark (1996), language is a joint activity that requires coordination between speaker and listener. Eye contact serves as a fundamental non-verbal cue, a social mechanism for attention that usually precedes executing behavior (e.g., speaking). As when calling someone’s name, making eye contact signals the intention to communicate and/or of social interaction (Brennan et al., 2008, 2010). The eye contact effect points towards the accounted phenomena that eye contact activates the social brain and modulates cognitive processing and/or behavioral response, so reflecting the social function of eye contact in human interaction (for a review, see Senju & Johnson, 2009). Furthermore, developmental studies suggest a predisposition among infants and newborns for making eye contact (Farroni et al., 2007; Morton & Johnson, 1991). There are parallels in the animal literature to help understand the role of eye gaze in human sociality and communication (Frith & Frith, 2023).

Reports on animal cognition literature also point towards a key social role of eye contact. Human eyes distinguish themselves from other primates in having wider horizontal elongation and uniform white sclera (Kano, 2023; Kobayashi & Kohshima, 1997; Luft et al., 2022; Mayhew & Gómez, 2015). Even though white sclera can be present in other mammals (Clark et al., 2023), primates and other evolutionary lineages have sclera that is pigmented or darker in color compared to human sclera (Kano, 2023; Kano et al., 2022). The ability of basic, reflexive gaze-based co-orientation may have evolved in fish and possibly may have been present as early as 325 million years ago in the stem amniote (Zeiträg et al., 2022). Eye characteristics have been shown to be sensible to adaptive changes. The evolutionary trait of uniformly unpigmented sclera in humans is understood to have adaptive values, for gaze perception, ostensive communication, and mostly for social interaction and social cognition (Clark et al., 2023; Kano et al., 2022; Yorzinski & Miller, 2020). The amount of white sclera in humans can be perceived as social cues of a person’s health, age, attractiveness, trustworthiness, emotional status, and aggressive threat (see (Wacewicz et al., 2022). Faces with whiter sclera are perceived by children as more cooperative than faces with darker sclera (Wolf et al., 2023). Faces with larger eyes (more visible sclera) are rated as being more attractive by adults and are fixated on for longer by 5-month-old babies (Geldart et al., 1999). Children and adults prefer stuffed animals with sclera that is visibly white over other colors (Segal et al., 2016). Thus, evolutionary drivers of white sclera in humans can be pivotal to shape human sociality. The human eye functions not only as a sensory organ, but as a signaling one as well (Kano, 2023; Wacewicz et al., 2022).

The human field of view comprises both central and peripheral vision. Central vision is directed towards the fovea, which represents the most sensitive part of the retina (Rehman et al., 2023). While central vision has the highest visual acuity, covering around 13 degrees and allowing one to fixate at one point at a time, peripheral vision has poor acuity but enables detection of color changes, contrasts, and motion. Rapid eye movements (saccades) dynamically shift our central vision between fixations so that we can see detail (Hessels, 2020; Younis et al., 2019). This characteristic in our vision, essentially the limited size of the fovea, has an effect on close, face-to-face, eye contact: We can only focus on the right eye of speakers, the left eye, or at a point in between – on the nose bridge – to capture both eyes peripherally.

In human social conversations, in addition to more fixations on both eyes of a speaker, differences have been reported on more fixations to the right eye (left visual field) than the left eye of the speaker (Barton et al., 2006; Eisenbarth & Alpers, 2011; Ma et al., 2022; Smith et al., 2013). This so-called left perceptual bias with the viewer focusing on the speaker’s right eye appears in a range of situations, including when freely viewing faces (Coutrot et al., 2016; Smith et al., 2013), recognizing faces (Barton et al., 2006; Ma et al., 2022; Peterson & Eckstein, 2013), rating emotions (Eisenbarth & Alpers, 2011; Thomas et al., 2014), and judging attractiveness and trustworthiness (Hermens et al., 2018; Thomas et al., 2014). The most plausible hypothesis for this perceptual asymmetry is a right hemispheric advantage for face processing (Yovel et al., 2008) and audiovisual speech perception (Baynes et al., 1994; Diesch, 1995; Smeele et al., 1998). It takes into account that sensory input from the left visual field projects onto the right hemisphere (Smith et al., 2013). Hence, this asymmetrical gaze behavior is commonly attributed to a perceptual (viewer) bias, rather than to intrinsic qualities of the viewed face, i.e., a hemifield rather than a hemiface bias (Barton et al., 2006; Everdell et al., 2007; Gilbert & Bakan, 1973; Mertens et al., 1993). This conclusion has been reached mostly due to studies showing that the left perceptual bias persists in experiments exposing individuals to stimuli such as chimeric images and mirrored faces (Everdell et al., 2007; Smith et al., 2013).

Given the social function of the eyes in humans and non-humans, we argue that the left perceptual bias theory might be too rudimentary. Considering the social role of eye gaze (primarily during non-speaking eye contact) and the fact that eye morphology (e.g., visible sclera, and wider elongation) marks a social function among humans and non-humans, two hypothetical predictions regarding the left perceptual bias emerge. First, we predict larger visible white sclera in one eye affects face scanning patterns and will consequently modulate the left perceptual bias itself (sclera modulation hypothesis). Second, we predict that the social function in the mode of communication (i.e., speaking vs. non-speaking) modulates the left perceptual bias (communication modulation hypothesis).

Two eye-tracking experiments implemented actual human faces (Experiment 1) and a virtual human face (Experiment 2) investigating the emergence of the left perceptual bias and the effect of eye-size asymmetries during both speaking and non-speaking conditions in light of the sclera modulation hypothesis and the communication modulation hypothesis.

## Methods

Two eye-tracking experiments recorded gaze behavior with university students who received partial course credit. The corresponding Research Ethics and Data Management Committee provided ethical clearance for both experiments (REDC 2019.03ab).

In Experiment 1 a total of 40 participants (mean 20.35 ± 2.07 years old; 22 male, 18 female) looked at 16 pre-recorded videos of a male and a female speaker presenting a problem, and answered questions. In Experiment 2 a total of 68 participants (mean 21.69 ± 3.12 years old; 19 male, 48 female, one other) watched 16 video animations of a female virtual human presenting a problem, and answered questions. Inclusion criteria were having a normal or corrected-to-normal vision, and having neither contact lenses nor cognitive, visual, auditory or neurological conditions.

The videos (i.e., experimental stimuli) had speakers telling semi-structured short stories containing six elements of information. They described a person travelling from one place (origin) to another (destination), at one specific day and time, with a specific purpose and using a specific mode of transportation. The duration of the videos was 12.76 ± 0.80 s in Experiment 1 and 11.46 ± 0.76 s in Experiment 2. They included an initial idle time (non-speaking), speaking period, and a final idle time (non-speaking). Idle times lasted 2.47 ± 0.26 s. At the end of each video, participants were asked a multiple-choice question about its content. The aim was to motivate the participant with a (short) memory task to maintain attention while watching the speaker. Videos in Experiment 1 presented speakers in their original form and a flipped version of the video, so that the face of the speaker appeared mirrored in the flipped versions. It allowed for two different eye-size asymmetries for each speaker (e.g., if the speaker has a larger right eye than left eye, the flipped version will appear as the reverse, with a larger left eye than right eye). In turn, Experiment 2 included videos presented in normal and manipulated conditions. While manipulation of the eyes suppressed blinking, manipulation of the mouth limited mouth motion throughout the video.

For the experimental procedure, participants arrived at the research lab and signed an informed consent form. Videos were displayed in a vertically oriented monitor that incorporated an eye-tracker device (SMI SensoMotoric Instruments) with a mounting bracket positioned at 20° visual angle. The eye-tracker recorded participants’ eye movements with a 60-Hz sampling frequency. A calibration at the beginning of the experiment and validation stages after presentation of every four videos were implemented. Calibration consisted of gaze-following a moving target on the screen to map the participant’s field of view according to eye motion. An introductory video that allowed the participant to become familiarized with (one of) the speakers presented further instructions. Thereafter the 16 videos were presented following a prior randomization procedure. All videos were counterbalanced so that participants watched four videos per condition in Experiment 1 (real and flipped videos, female and male speakers) and two videos per condition in Experiment 2 (conditions included manipulation of mouth, manipulation of eyes, and no manipulation).

Three areas of interest (mouth and left and right eyes) were defined using dedicated software (BeGaze 3.7). A semiautomatic procedure dynamically shifted areas of interest along the video timeline, with occasional manual corrections. Fixation was defined as holding the gaze on one area of interest for at least 80 ms (Hessels et al., 2018). The analyzed dependent variable set according to the research questions was accumulated fixation time, which provided an averaged fixation pattern by merging data from all participants and stimuli, and allowed for comparisons across three time chunks of stimulus time (initial non-speaking, speaking, and final non-speaking). Additionally, we implemented a ratio parameter to calculate the proportion of fixations between the eye and mouth along stimulus time, for both the left and the right eyes.

To estimate eye size, we approached the eye as if having an ellipse shape using the equation of the area of an ellipse, *A* = *πab*, where ‘a’ and ‘b’ are the minor and major ellipse ratios, respectively. Eye width and eye height were calculated (in pixels) by drawing lines in both eyes over still images taken from the initial idle times (i.e., before speakers in the videos actually begin to speak) using graphics software. The lines represented the longest vertical and horizontal distances in each eye, using as reference the borders of the sclera. Our procedure for definition of eye width and height thus followed the definitions reported by the Chicago face database (Ma et al., 2015).

Statistical analyses were conducted in RStudio 2024.04.2 + 764. We compared fixation parameters across areas of interest (left eye, right eye, and mouth) using the linear mixed-effects model afex package (Singmann et al., 2023). Random factors included both participants and items. A time analysis divided stimulus time in initial non-speaking (idle time), speaking, and final non-speaking times (idle time). Least-square means using the emmean package and based on z-test asymptotic methods were used for post hoc comparisons (Lenth, 2023).

## Results

When participants looked at human speakers, fixations towards the mouth and eyes differed, *F*(2, 35,671.72) = 25,821.80*, p* <.01, with gaze directed at both the right and the left eyes being longer than gaze directed at the mouth, *z* = −225.68, *p* < *.*01, and *z* = −134.25, *p* < *.*01, respectively.

Fixation towards the eyes, specifically, was larger during non-speaking (idle times) in comparison to speaking events. When compared to speaking time, as predicted by the communication modulation hypothesis, right eye fixations were significantly longer during initial non-speaking time, *z* = 42.55, *p* < *.*01, and final non-speaking time, *z* = 65.90, *p* < *.*01. The same was true for fixations in the left eye of the speaker being larger during both initial, *z* = 51.32, *p* < *.*01, and final non-speaking times, *z* = −55.78, *p* < *.*01 than during speaking.

**Fig. 1:**
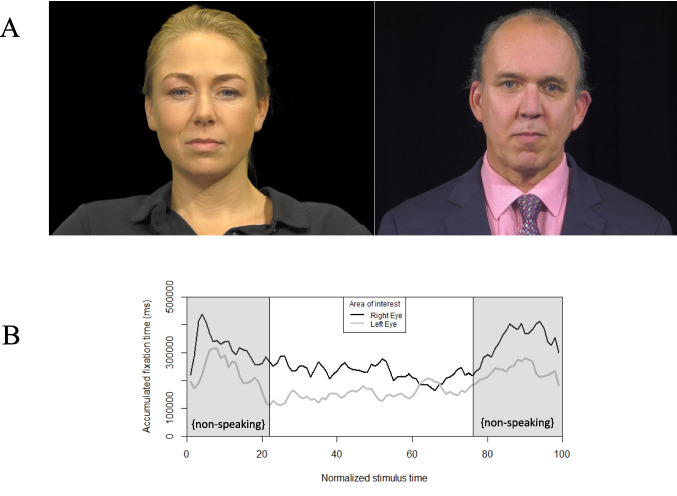
**A** Stimuli in Experiment 1: male and female human speakers. (**B**) Fixation time towards the left and right eyes during non-speaking (gray phases) and speaking conditions. Stimuli had an average total duration of 12.76 ± 0.80 s

As predicted by the left perceptual bias hypothesis, fixations on the left and the right eye of the speaker were not equally distributed. A bias was found for directing gaze towards the right eye of the speaker (i.e., towards the reader and the participants’ left visual field), more than the left eye, *z* = 110.63, *p* < *.*01. Figure 1 shows the female and male human speakers used in Experiment 1 and the accumulated fixation over stimulus time, comparing gaze directed at left and right eyes, and fixations during speaking and non-speaking times.

**Fig. 2:**
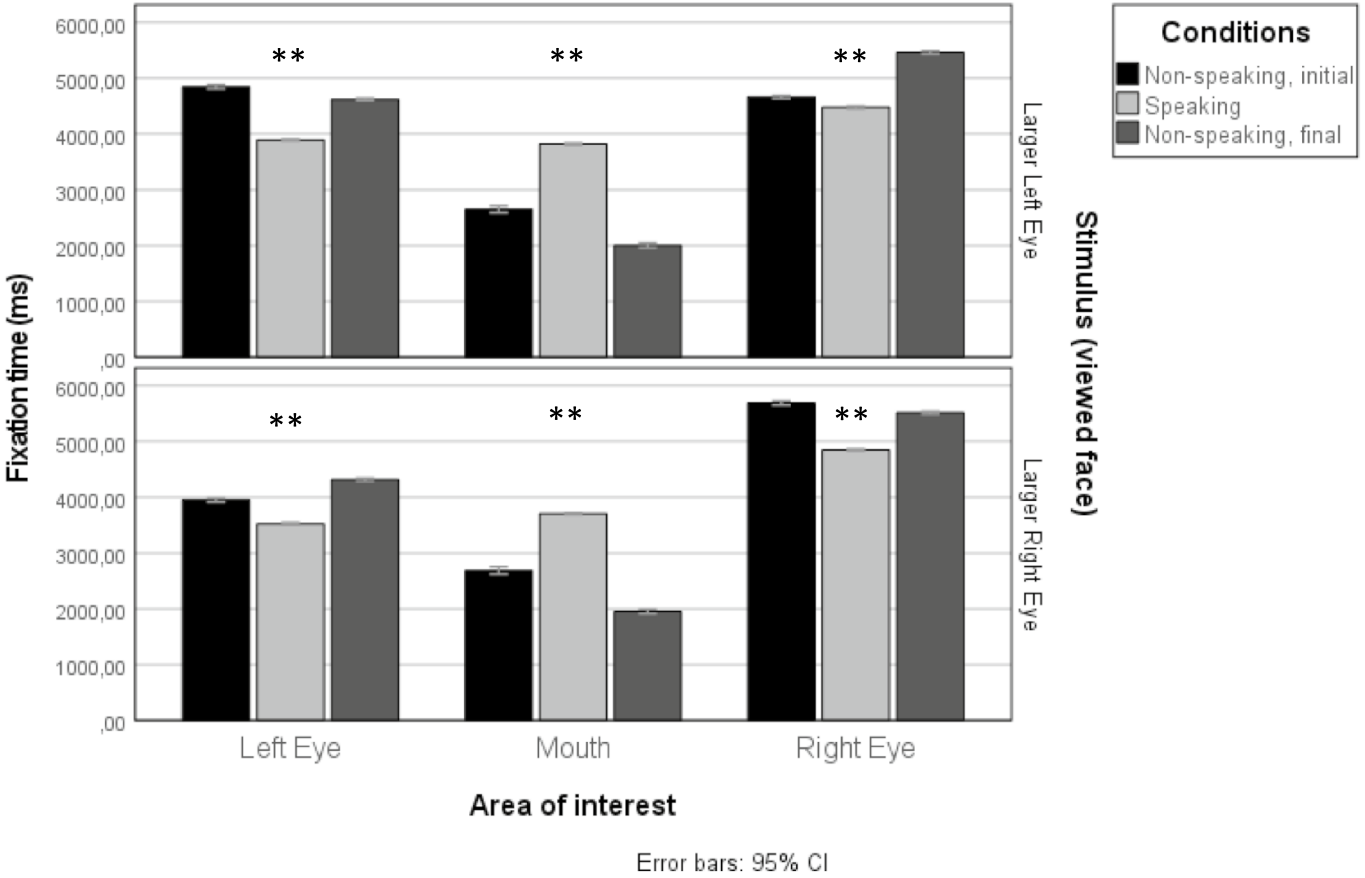
Fixation time towards left and right eyes and mouth during non-speaking and speaking conditions in human face stimuli with an apparent larger left eye (**upper panel**) and a larger right eye (**lower panel**). Whereas fixations to the mouth increase during speaking conditions, fixations to the eyes decrease. ** *p* <.01 in the comparison between speaking and non-speaking periods

Humans have eye-size asymmetries, but not predictably for one eye (Ma et al., 2015). Whereas the *right* eye of the male narrator was 7.88% larger than his left eye, the female narrator had a *left* eye 8.44% larger than her right eye. We next analyzed separately fixations from videos where the speaker has a larger right eye (i.e., the original version of the male speaker and the flipped version of the female speaker), and fixations from videos where the speaker has a larger left eye (i.e., the flipped version of the male speaker and original version of the female speaker) (Fig. 2).

**Fig. 3:**
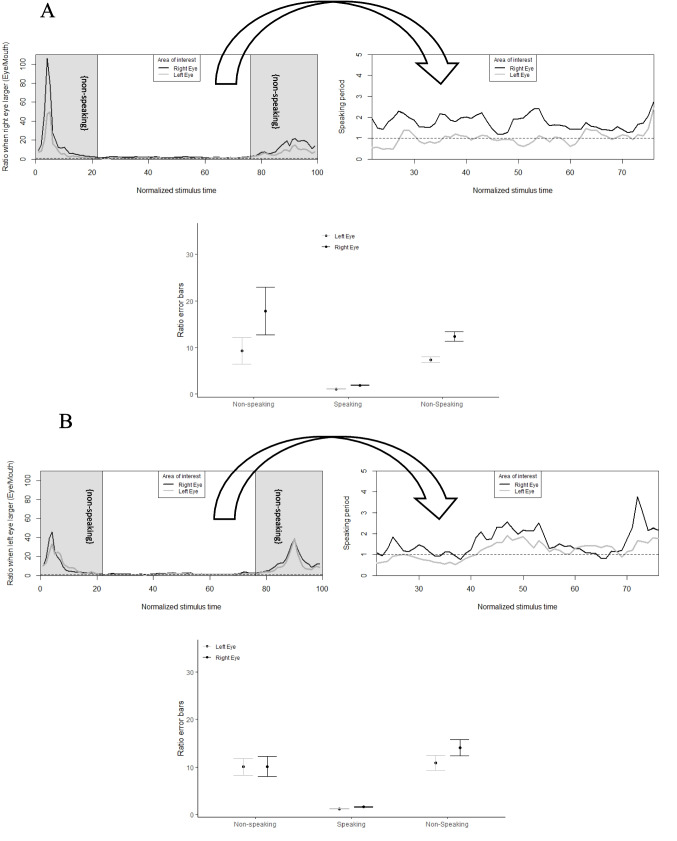
Ratio of fixations (eye/mouth) towards humans with (**A**) an apparent larger right eye and (**B**) an apparent larger left eye. Ratio of fixations above and below the dashed horizontal line at 1 indicate larger eye fixations and larger mouth fixations, respectively. Error bars (bottom panel) are clustered according to non-speaking and speaking conditions

A ratio parameter that estimated the proportion of fixations between eye and mouth along stimulus time showed participants directing their gaze towards the right eye more than to the left eye, *t*(390) = −3.32, *p* <.01 (Fig. 3, upper panel), again supporting the left perceptual bias. On the other hand, speakers with a larger left eye did not yield a difference in ratio fixations between left and right eyes, *t*(390) = −1.37, *p* = *.*17 (Fig. 3, lower panel), and therefore the left perceptual bias was not confirmed. These findings are in line with the sclera modulation hypothesis.

As predicted by both the sclera modulation and the communication modulation hypotheses, we have so far shown that the right eye bias takes place mostly in non-speaking conditions and when the apparent right eye is larger than the left eye. These findings suggest that the right eye bias (1) is not purely perceptual, and (2) can be modulated by inner features in the viewed face. To further investigate such implications, a second experiment exposed participants to a virtual human in normal and manipulated conditions, whereby we were able to restrict the movement of the agent’s eyes and mouth.

**Fig. 4:**
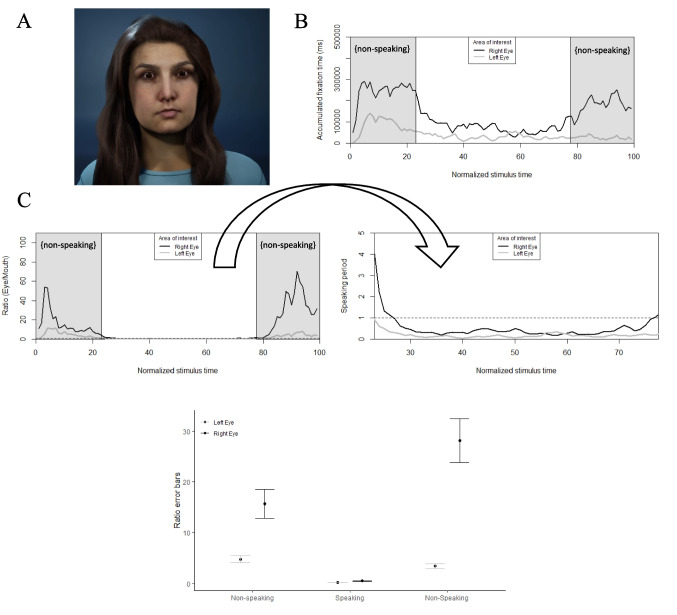
**A** Stimuli in Experiment 2: female virtual human. (**B**) Fixation time towards left and right eyes during non-speaking (gray phases) and speaking conditions. Stimuli had an average duration of 11.46 ± 0.76 s, which included an idle (non-speaking) time of 2.47 ± 0.26 s both before and after (gray phases) the speaking period. (**C**) Ratio of fixations. Values above and below the dashed horizontal line at 1 indicate larger eye fixations and larger mouth fixations, respectively. Error bars (bottom panel) are clustered according to non-speaking and speaking conditions

Fixation patterns towards a virtual human mimicked the findings for human speakers (Fig. 4). Consistent with observations in actual humans, there was a main effect on fixations across areas of interest when looking at a virtual human (left and right eyes and mouth), *F*(2, 2643.72) = 6633.76, *p* < *.*01. Stimulus time including speaking and non-speaking periods also had an effect on fixation time, *F*(2, 8556.00) = 499.00, *p* < *.*01. Pairwise comparisons showed that participants had a bias to fixate more at the right eye than at the left eye during (non-speaking) initial, *z* = 71.41, *p* < *.*01, and final idle times, *z* = 74.02, *p* < *.*01, as well as during speaking time, *z* = 43.34, *p* < *.*01. This is consistent with the sclera modulation hypothesis because the virtual human had a right eye 13.93% larger than her left eye.

Additionally, eye-to-mouth fixation ratios show a redistribution of face-scanning patterns during speaking conditions consistent with the communication modulation hypothesis. Specifically for fixations towards the right eye, the ratio decreased from initial idle time to speaking period, *t(192)* = 7.39, *p* <.01, and increased during the final idle time in comparison to both the speaking period, *t(192)* = *-*13.25, *p* <.01, and initial idle time, *t(192)* = −5.06, *p* <.01. The ratio of fixations between left eye and mouth did not change significantly across speaking and non-speaking conditions, *p* >.05.

**Fig. 5:**
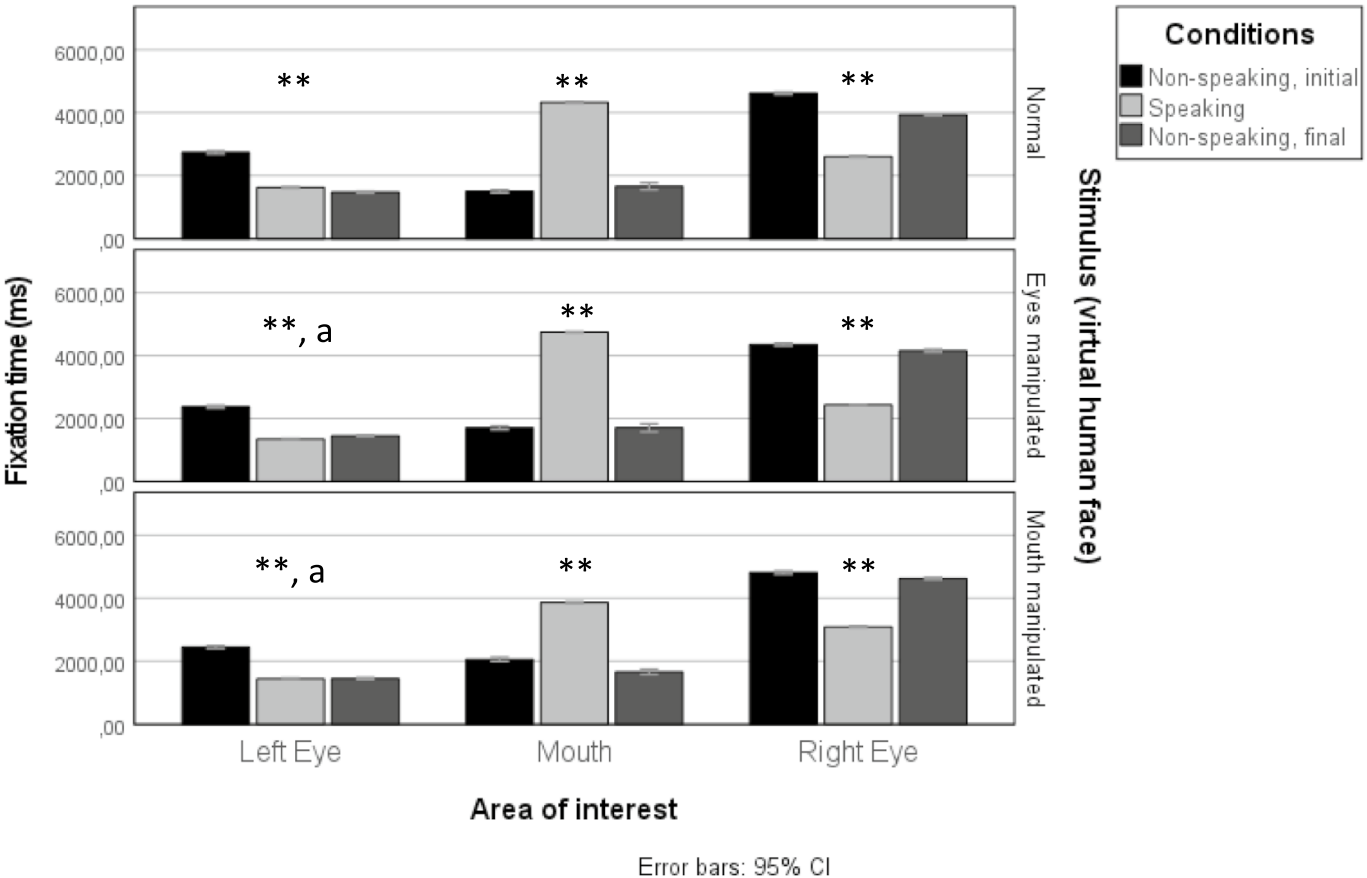
Fixation time towards left and right eyes and mouth during non-speaking and speaking conditions in a virtual human face with an apparent larger right eye, during normal (**upper panel**), eyes manipulated (**middle row**), and mouth manipulated (**lower panel**) conditions. ** *p* <.01 in comparison between speaking and non-speaking periods, with exceptions in ‘a’ where no statistical difference was found between speaking and final non-speaking periods

To investigate further whether the bias to fixate on the right eye is purely perceptual or not, we manipulated inner facial cues in the virtual human. In the mouth manipulated condition, lip movement was immobilized. In the eye manipulation blinking was removed (Fig. 5).

**Fig. 6:**
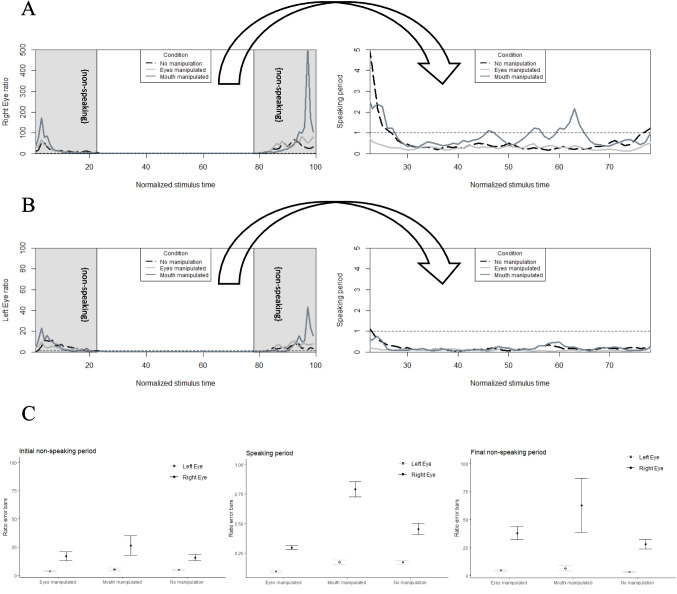
Ratio of fixations towards (**A**) the right eye and (**B**) the left eye of a virtual human along stimulus time, showing non-manipulated and manipulated conditions. Gray phases mark initial and final non-speaking periods. Right panels zoom in the speaking period. Ratio of fixations above 1 (dashed horizontal line in upper panels) indicate larger eye fixations and values below indicate larger mouth fixations. (**C**) Error bars clustered according to non-speaking and speaking periods

As predicted by the sclera modulation hypothesis, fixation ratios towards the right eye were larger than to the left eye, *t*(576) = −8.14, *p* <.01. Additionally, as predicted by the communication modulation hypothesis, participants had stronger fixation ratios towards the right eye during non-speaking conditions when the virtual human presented with a manipulated mouth, in comparison to a normal (i.e., non-manipulated) virtual human, *t(576)* = −4.69, *p* <.01. Meanwhile, ratio fixations towards the left eye did not yield the same significant differences across normal and manipulated conditions, *p* >.05. The latter results suggest that in addition to eye-size asymmetries, divergent inner facial cues such as immobilized mouth movements modulate the right eye bias. As with the human speakers, findings from agent faces also suggest that the right eye bias is not just perceptual (Fig. 6).

## Discussion

Eyes are for both social and communicative contact in face-to-face conversations. Two eye-tracking experiments found that not only did participants focus more on the eyes, they particularly focused on the eyes when the conversational partner did not speak. In addition, in line with prior literature, during eye contact the gaze of a participant demonstrated a left visual bias to fixate at the right eye of the speaker. However, this bias was affected by the social function of eye contact in a manner consistent with both the sclera and the communication modulation hypotheses. The social function of eye contact refers to a well-documented literature that highlights eye gaze as a fundamental social cue, used during communication as a mechanism of attention, coordination, and behavioral intent (Brennan et al., 2008, 2010; Clark, 1996; Frith & Frith, 2023; Senju & Johnson, 2009). In the present study, when the right eye of the speaker showed more sclera and when a speaker was not speaking, the left perceptual bias was reinforced, consistent with the social function from eye gaze. Meanwhile, when the left eye of the speaker showed more sclera and when the speaker is speaking, the left perceptual bias was mitigated. Additionally, the manipulation of mouth movements in a virtual human also reinforced the bias to fixate more at the right eye during non-speaking conditions, and decreased fixations to the mouth during speaking conditions. The latter can be partly explained by visual and motor salience (Butcher et al., 2025), particularly the incongruent (lack of) movement in the mouth. However, we believe that in real and dynamic human interactions, when facial features diverge from normal (i.e., socially expected) conditions, the eyes can serve as a social anchor and draw attention away from the mouth. Such a resolution could minimize distraction and facilitate (continued) effective communication. Hence, because of the modulation triggered by eye-size asymmetries, by non-speaking versus speaking conditions, and by manipulated mouth motion, our results further suggest that the left perceptual bias on the right size of the face is more than merely perceptual.

Our findings are in line with the sclera modulation hypothesis that takes into consideration the evolutionary social drivers of white sclera among humans, and by the communication modulation hypothesis that incorporates the social function of the eye. Thus, the present study refutes the idea that gaze strategies to look at faces respond merely to perceptual mechanisms. Instead, it implies that asymmetric face traits, different action ladders of communication, and unnatural facial cues modulate the right-eye bias.

Asymmetries between the left and right eyes are common. The modulatory effect from eye-size asymmetries on gaze behavior might be related to the social role of the eyes in humans. Some authors suggest that larger eye elongation and white sclera may have evolved to optimize gaze behavior, further distinguishing human eyes from great ape eyes (Mayhew & Gómez, 2015), a trait that could contribute significantly to amplifying gaze and for transmission of eye-based social cues, particularly during eye contact (Mayrand et al., 2023). In addition to perceptual lateralization, humans also demonstrate specialized motor lateralization. For instance, when we speak, the right side of the mouth moves significantly more than the left side (for a summary of studies, see Everdell et al., 2007), and most people are right-eye as well as right-foot and right-hand dominant (Coren, 1993). We speculate that lateralized motor articulation and expression in one side, and asymmetrical perceptual behavior in the other, carry an interconnected perception and action dynamic possibly assisted by hemispheric specialization. Thus, a perceptual bias to direct our gaze to our left visual field may represent a learning-by-experience strategy that responds to the larger amount of information guided by the larger motor input coming from the right side of the face. Such a mechanism would likely contribute to more efficient social interaction and communication among humans.

Given the sensory and signaling properties of the eyes (Wacewicz et al., 2022), we find it more appropriate to study the dynamic bias to the right eye as an unfolding psychological interaction that takes place in social communication, particularly during eye contact, and shaped by cognitive contextual situations such as relationship and level of confidence towards our conversational partners (e.g., hierarchical, friendly, intimate), and content of speech (e.g., personal).

The human facial stimuli in our study comprised only two levels of eye-size asynchrony and associated sclera visibility, which limits the generalizability of the findings across a broader spectrum of eye-size asynchrony observed in diverse and representative human faces. Therefore, the modulatory effect that faces with larger (sclera visibility on) left eyes have on the left perceptual bias requires further investigation. Additionally, our experimental design did not involve active social interaction, real face-to-face communication, or reciprocal eye contact. Instead, the experimental paradigm approximated a conversational context through passive video watching that simulated a communicative exchange: participants viewed (human) faces in idle and speaking conditions and subsequently responded to a question about the speaker’s narrated story. Thus, we acknowledge a limit in generalizing our results towards naturalistic, real-world social interactions. Moreover, eye size was estimated based on a series of static images taken from all stimuli, without accounting for dynamic features that can potentially impact the asynchrony of sclera visibility between the eyes. Further research can investigate how dynamic features possibly affect the asynchrony of sclera visibility during active social communication.

Furthermore, our design did not control for perceptual or motor salience (Butcher et al., 2025), or low-level visual features such as eye luminance or brightness contrast. Although we believe they may have a contributory role in face-scanning patterns (see, e.g., results from manipulated conditions when using a virtual human face as stimulus), we do not expect such factors to play a major role on eye fixations during real face-to-face interactions, in comparison to the modulatory social and communicative dynamics of perception and action driven by eye contact. Lastly, since emotional context can guide gaze fixations (Vetter et al., 2019), future studies may investigate the role of emotional faces during active social interactions.

Here we provided evidence that eye-size asymmetries, unnatural inner facial movements, and contextual tasks such as (silent) eye contact and speech processing modulate the steadily reported gaze behavior bias to look at the right hemiface and at the right eye of other people faces over their left eyes, a face-scanning behavior persistently described in the literature as the “left perceptual bias.” By incorporating human and virtual human stimuli during speaking and non-speaking conditions, our results partially support previous studies by reporting a preferred strategy to fixate at the right eye (i.e., towards the left visual field from the viewer perspective). However, our findings extend prior research by further showing that inner facial features fine-tune this bias so that we look more at larger eyes (meaning that the hemiface where the larger eye is matters), and that abnormal mouth movements can exacerbate the right-eye bias following speech processing. Finally, we show that the right-eye bias takes place mostly in non-speaking conditions, for example during eye contact.

All in all, these findings shed light on the most natural form of human–human communication, that of face-to-face interactions, informing researchers of evolutionary psychology, cognitive psychology, and the communication and computer sciences alike.

## Data Availability

The code used in this study will be made available upon reasonable request.

## Declarations

### Open practices statement

The datasets generated and/or analyzed during the current study are available from the authors on reasonable request.
